# Long-Term Trends in Esophageal Candidiasis Prevalence and Associated Risk Factors with or without HIV Infection: Lessons from an Endoscopic Study of 80,219 Patients

**DOI:** 10.1371/journal.pone.0133589

**Published:** 2015-07-24

**Authors:** Yuta Takahashi, Naoyoshi Nagata, Takuro Shimbo, Takeshi Nishijima, Koji Watanabe, Tomonori Aoki, Katsunori Sekine, Hidetaka Okubo, Kazuhiro Watanabe, Toshiyuki Sakurai, Chizu Yokoi, Masao Kobayakawa, Hirohisa Yazaki, Katsuji Teruya, Hiroyuki Gatanaga, Yoshimi Kikuchi, Sohtaro Mine, Toru Igari, Yuko Takahashi, Akio Mimori, Shinichi Oka, Junichi Akiyama, Naomi Uemura

**Affiliations:** 1 Department of Gastroenterology and Hepatology, National Center for Global Health and Medicine, Tokyo, Japan; 2 Ohta Nishinouchi Hospital, Fukushima, Japan; 3 Division of AIDS Clinical Center (ACC), National Center for Global Health and Medicine, Tokyo, Japan; 4 Department of Pathology, National Center for Global Health and Medicine, Tokyo, Japan; 5 Division of Rheumatic Diseases, National Center for Global Health and Medicine, Tokyo, Japan; 6 Department of Gastroenterology and Hepatology, National Center for Global Health and Medicine, Kohnodai Hospital, Chiba, Japan; University of Wisconsin Medical School, UNITED STATES

## Abstract

**Background:**

The prevalence of candida esophagitis (CE) might be changing in an era of highly active antiretroviral therapy (HAART) among HIV-infected patients or today’s rapidly aging society among non-HIV-infected patients. However, few studies have investigated long-term CE trends, and CE risk factors have not been studied in a large sample, case-control study. This study aimed to determine long-term trends in CE prevalence and associated risk factors for patients with or without HIV infection.

**Methods:**

Trends in CE prevalence were explored in a cohort of 80,219 patients who underwent endoscopy between 2002 and 2014. Risks for CE were examined among a subcohort of 6,011 patients. In risk analysis, we assessed lifestyles, infections, co-morbidities, immunosuppressants, and proton-pump inhibitors (PPIs). All patients were tested for HIV, hepatitis B or C virus, and syphilis infection. For HIV-infected patients, sexual behavior, CD4 cell count, history of HAART were also assessed.

**Results:**

CE prevalence was 1.7% (1,375/80,219) in all patients, 9.8% (156/1,595) in HIV-infected patients, and 1.6% (1,219/78,624) in non-HIV-infected patients. CE prevalence from 2002-2003 to 2012-2014 tended to increase in non-HIV-infected patients (0.6% to 2.5%; P<0.01) and decrease in HIV-infected patients (13.6% to 9.0%; P=0.097). Multivariate analysis revealed increasing age (odds ratio [OR], 1.02; p=0.007), HIV infection (OR, 4.92; p<0.001), and corticosteroid use (OR, 5.90; p<0.001) were significantly associated with CE, and smoking (OR, 1.32; p=0.085) and acetaminophen use (OR, 1.70; p=0.097) were marginally associated. No significant association was found with alcohol consumption, hepatitis B or C virus, syphilis, diabetes mellitus, cardiovascular disease, cerebrovascular disease, chronic kidney disease, liver cirrhosis, anticancer, or PPIs use. In HIV-infected patients, CD4 cell count <100/μL (OR, 4.83; p<0.001) and prior HAART (OR, 0.35; p=0.006) were independently associated with CE, but sexual behavior was not. Among corticosteroid users, CE was significantly associated with higher prednisone-equivalent dose (p=0.043 for trend test).

**Conclusions:**

This large, endoscopy-based study demonstrated that CE prevalence increased in non-HIV-infected patients but decreased in HIV-infected patients over 13 years. Risk analysis revealed that increasing age, HIV infection, and corticosteroids use, particularly at higher doses, were independently associated with CE, but alcohol, other infections, diabetes, anticancer drugs, and PPIs use were not.

## Introduction

Candida esophagitis (CE), one of the most common opportunistic infections of the esophagus [1], is classed as an invasive fungal disease [2]. Severe CE may cause esophageal hemorrhage or progress to stricture or fistula formation, accompanied by reduced quality of life. Early diagnosis and treatment with systemic antifungals is therefore important [3]. Endoscopy is an essential diagnostic tool for detecting fungal white plaque and performing biopsy [4], but it is too costly and invasive to be used in an unselected population. Therefore, high-risk patients must be identified to prevent delays in diagnosis and poor outcomes.

Several studies have reported that proton-pump inhibitors (PPIs), immunosuppressants, and HIV infection are common risk factors for CE, although most of these studies did not have a case-control design, had a small samples, or were restricted to immunocompromised patients [5–8]. In today’s rapidly aging society, it is unclear whether these risk factors remain the same given that comorbidities in general are expected to rise with rapid aging of the population [9] and the suggested risk factors of PPIs and immunosuppressant drugs [6,8,10] are now in widespread clinical use. In contrast, it is known that HIV-related opportunistic infections have decreased dramatically following the introduction of highly active antiretroviral therapy (HAART) [11,12]. Moreover, few studies have investigated CE risk factors in the recent decade and long-term trends in CE prevalence have not been studied to date [7,10]. In light of such societal changes, the risk factors for CE should be reconsidered and trends in CE prevalence investigated to facilitate timely diagnosis and treatment of the disease.

Here we report the results of a large, endoscopy-based, cross-sectional, case-control study conducted over 13 years that aimed to identify trends in annual CE prevalence in patients with or without HIV infection and determine through risk analysis which lifestyle factors, medication, and comorbidities are associated with CE.

## Materials and Methods

### Study design, setting, and participants

We conducted two studies at the endoscopy unit of the National Center for Global Health and Medicine (NCGM; Tokyo, Japan) between January 2002 and April 2014, one to investigate CE prevalence and the other to examine risk factors for CE. This study was approved by the ethics committee of the National Center for Global Health and Medicine Center (Nos. 711, 1424) and was implemented in accordance with the provisions of the Declaration of Helsinki. NCGM has 900 beds and is the largest referral center for HIV/AIDS in Japan. All patients underwent serological testing for HIV, hepatitis B virus (HBV), hepatitis C virus (HCV), and syphilis before endoscopy [13]. Written informed consent was obtained from all patients prior to endoscopy.

In Study 1 to identify overall CE prevalence and trends in annual CE prevalence, we reviewed a hospital-based, prospectively collected endoscopic database of 80,360 consecutive patients who underwent upper endoscopy at NCGM between January 2002 and April 2014. Indications for endoscopy were as follows: (i) continual or intermittent upper GI symptoms; (ii) asymptomatic with anemia; (iii) asymptomatic requiring examination for specific diseases due to abnormal findings on tumor marker or fecal occult blood testing or on abdominal imaging; (iv) screening required for gastric cancer; and (v) therapeutic endoscopy required. In Japan, which has a high incidence of gastric cancer [14], endoscopy is frequently performed for gastric cancer screening [15]. We excluded patients who were ≤18 years of age or who did not provide informed consent. All inclusion and exclusion criteria were fullfilled before the patients were enrolled.

In Study 2 to identify CE risk factors, we conducted a hospital-based, case-control study at the endoscopy unit of the NCGM between September 2009 and April 2014. Inclusion criteria were Japanese nationality and the aforementioned indications for endoscopy. Exclusion criteria were as follows: (i) no informed consent obtained; (ii) unknown use of medication; (iii) not independent in activities of daily living (ADL); (iv) inability to understand written documents; (v) use of any anti-fungal drugs within 1 month before endoscopy; (vi) urgent or early endoscopy for acute GI bleeding; and (vii) second endoscopy required during the study period. All inclusion and exclusion criteria were fullfilled before the patients were enrolled. The control group comprised 5,800 patients undergoing endoscopy during the same period who did not meet our criteria for the diagnosis of CE.

### Data sources and measurement

For risk analysis, histories of HBV, HCV, and syphilis infection were collected before endoscopy and defined as presence of antibody against hepatitis B or C surface antigen and positive results on a *Treponema pallidum* hemagglutination test, respectively, as previously reported [13].

A detailed questionnaire was also completed at the endoscopy unit on the same day as before endoscopy [16]. Well-trained medical researchers asked patients about alcohol consumption, smoking status, comorbidities, and medication use. Researchers also checked prescriptions and medical records in addition to the information provided by the patients to avoid omissions. Alcohol consumption was defined as consumption of alcohol at least once a week. Smoking was defined as currently smokes or ever smoked. Comorbidities included diabetes mellitus (DM), cardiovascular disease, cerebrovascular disease, chronic kidney disease (CKD), liver cirrhosis, peptic ulcer disease, post-esophageal resection, and post-gastric resection. DM was considered present in patients taking anti-diabetes drugs. CKD was considered present in patients on hemodialysis or peritoneal dialysis, or with serum creatinine levels ≥2.0 mg/dl. Patients were asked to indicate which drugs, if any, they had used based on drugs pictured in the questionnaire, as previously reported [17,18]. Use of a drug was defined as intermittent or regular administration within 1 month before the questionnaire interview. Patients were asked about the following medication use: anticancer drugs for solid or hematological tumors; 5 types of systemic corticosteroids (prednisone, methylprednisolone, betamethasone, dexamethasone, and hydrocortisone) and daily corticosteroid dose; 8 types of non-steroidal anti-inflammatory drugs (NSAIDs); acetaminophen; 4 types of PPIs (lansoprazole, rabeprazole, omeprazole, and esomeprazole); and 6 types of H2RAs (nizatidine, roxatidine, famotidine, ranitidine, lafutidine, and cimetidine). We calculated prednisone-equivalent daily corticosteroid dose based on the data that glucocorticoid doses providing anti-inflammatory effects approximately equivalent to 5 mg prednisone are methylprednisolone 4 mg, betamethasone 0.75 mg, dexamethasone 0.75mg, and hydrocortisone 20 mg [19].

For HIV-infected patients, routes of infection were determined using questionnaires administered by medical staff [20] and classified into one of six categories: homosexual, bisexual, heterosexual, drug user, use of untreated blood products, or unknown. Sexual behavior was divided into two categories: men who have sex with men (MSM) and heterosexual. Patients who were neither homosexual nor bisexual were regarded as heterosexual. CD4 cell count values obtained within 1 month before or after endoscopy were obtained from the medical records.

### Diagnosis of upper GI disease and candida esophagitis

**Fig 1:**
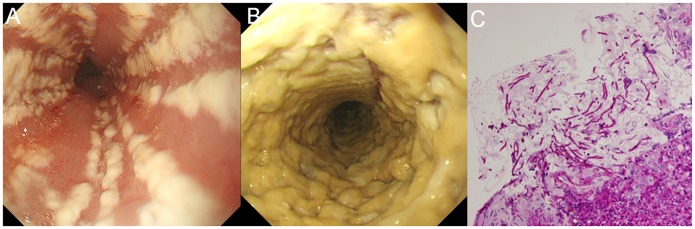
Endoscopic findings (A, B) and pathological findings (C) of candida esophagitis. (A) Confluent, linear, and nodular elevated plaques. (B) Thick white plaque cover on esophageal mucosa circumferential narrowing the lumen. (C). Numerous Candida pseudohyphae and spores in the exfoliated esophageal epithelium and detached superficial squamous epithelium (×400).

A high-resolution scope (GIF-H260, Olympus Corp., Tokyo, Japan) was used for diagnosis of upper GI diseases. Well-trained staff performed endoscopy while blinded to the questionnaire results. When abnormal findings were detected on endoscopy, biopsy or endoscopic mucosal resection was performed. All removed specimens were evaluated by expert pathologists (>10 years of experience), and final diagnoses of upper GI diseases were made. Diagnosis of CE was made on the basis that candida white plaques in the esophagus, detected by endoscopy, cannot be washed away [21] (Fig 1A and 1B) and confirmation by pathological assessment with hematoxylin and eosin and periodic acid-Schiff staining (Fig 1C) or culture for *Candida* species [17,18].

### Statistical analysis

In Study 1, we examined trends in CE prevalence over the 13-year study period in all subjects, HIV-infected patients, and non-HIV-infected patients, assessing changes in prevalence in 2-year bins with the Chi-squared test for linear trends.

In Study 2, we summarized descriptive data for patients with and without CE to identify risk factors. Patient characteristics were compared using the Mann-Whitney U test for quantitative variables or the Chi-square test or Fisher’s exact test for qualitative variables. The association between CE and other upper GI diseases was evaluated from both the endoscopy and pathology findings. CE risk factors were identified using a multiple logistic regression model including factors with a significance of P<0.2 on univariate analysis. Odds ratios and 95% confidence intervals were calculated. CE risk factors were also evaluated separately for HIV-infected and non-HIV-infected patients.

A *P* value of less than 0.05 was considered statistically significant. All statistical analyses were performed using Stata version13 software (StataCorp, College Station, TX).

## Results

### Study 1: Trends in CE Prevalence

**Fig 2:**
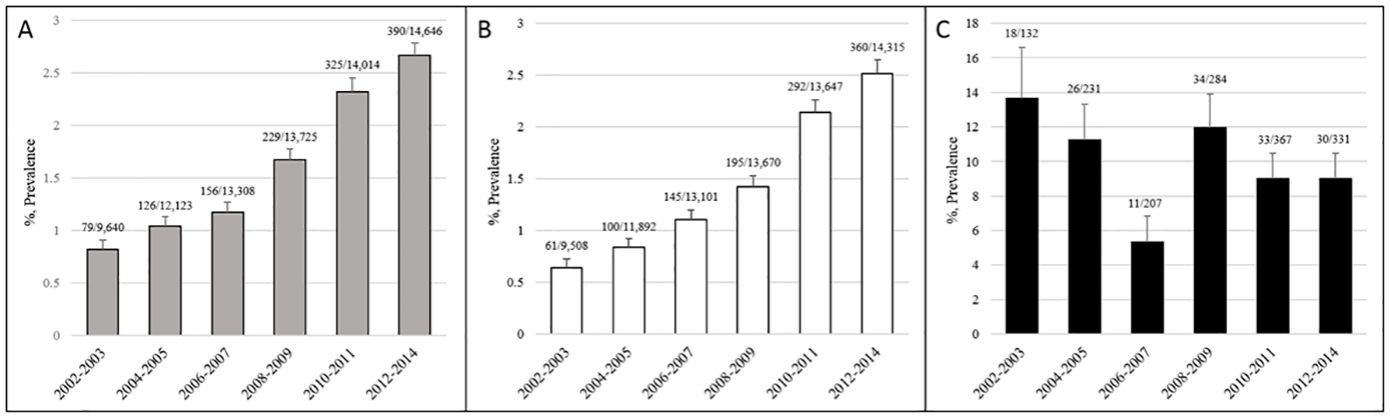
Trends in prevalence of candida esophagitis, 2002–2014. (A) All subjects, (B) non-HIV-infected patients, (C) HIV-infected patients. Values on bars are number of subjects testing positive/total number tested. Error bars show 95% confidential intervals.

Between January 2002 and April 2014, 80,360 patients underwent upper endoscopy at NCGM. After exclusion, 80,219 patients were left for analysis. CE prevalence was 1.7% (1,375/80,219) in all patients, 9.8% (156/1,595) in HIV-infected patients, 1.6% (1,219/78,624) in non-HIV-infected patients, and 1.8% (785/42,555) in patients aged >65 years. CE prevalence from 2002–2003 to 2012–2014 tended to increase in all subjects (0.8% to 2.7%, P<0.01 by trend test) (Fig 2A) and in non-HIV-infected patients (0.6% to 2.5%, P<0.01 by trend test) (Fig 2B), but decrease in HIV-infected patients (13.6% to 9.0%, P = 0.097 by trend test) (Fig 2C).

### Study 2: Risk factors for CE

#### Patient characteristics

Between September 2009 and April 2014, among the 32,827 patients who underwent upper endoscopy, we did not conduct questionnaire interviews with 23,485 patients because they either did not provide informed consent, did not know what medications they were receiving, were not independent in ADL, or could not understand written documents, and we found 3,331 patients met one of the exclusion criteria. This left a subcohort of 6,011 patients who underwent endoscopy and completed the questionnaire interview for analysis of CE risk.

Baseline characteristics are shown in Table 1. The factors significantly associated with CE were alcohol consumption, HIV infection, CD4 cell count, history of HAART, HCV infection, syphilis, and use of corticosteroids, NSAIDs, acetaminophen, or PPIs. As shown in Table 2, the upper GI diseases significantly associated with CE were esophageal ulcer, gastric ulcer, and severe atrophic gastritis.

**Table 1:** Patient characteristics in the entire cohort and groups with or without candida esophagitis.

	All (n = 6,011)	CE (n = 211)	Non-CE (n = 5,800)	P*
Age, years	59.6±14.2	60.3±15.0	59.6±14.2	0.469
Sex, male	3,397 (56.5)	128 (60.7)	3,269 (56.4)	0.216
Alcohol	3,185 (3.0)	97 (46.0)	3,088 (53.2)	0.038
Smoker	2,758 (45.9)	110 (52.1)	2,648 (45.7)	0.064
Infection				
HIV infection	430 (7.2)	48 (22.8)	382 (7.6)	<0.001
Infectious route				
Homosexual	255 (59.3)	30 (62.5)	225(58.9)	0.632
Bisexual	41 (9.5)	7 (14.6)	34 (8.9)	0.206
Heterosexual	67 (15.6)	6 (12.5)	61 (16.0)	0.532
Drug user	2 (0.5)	0 (0)	2 (0.5)	0.615
Untreated blood products	46 (10.7)	2 (4.2)	44 (11.5)	0.120
unknown	19 (4.4)	3 (6.3)	16 (4.2)	0.512
CD4 cell count/μL, median (IQR)	405 (222, 584)	143 (30, 403)	432 (262, 597)	<0.001
CD4 cell count/ <100/μL	65 (15.1)	23 (47.9)	42 (11.0)	<0.001
History of HAART	342 (79.5)	24 (50.0)	318 (83.3)	<0.001
Duration of HAART (months)	76.4±60.9	68.0±71.6	77.1±60.0	0.212
HBV infection	130 (2.2)	3 (1.4)	127 (2.2)	0.451
HCV infection	339 (5.6)	20 (9.5)	319 (5.5)	0.014
Syphilis	172 (2.9)	18 (8.5)	154 (2.7)	<0.001
Comorbidities				
Diabetes mellitus	783 (13.0)	25 (11.9)	758 (13.1)	0.605
Cardiovascular disease	444 (7.4)	16 (7.6)	428 (7.4)	0.912
Cerebrovascular disease	28 (0.5)	1 (0.5)	27 (0.5)	0.986
Chronic kidney disease	191 (3.2)	5 (2.4)	186 (3.2)	0.496
Liver cirrhosis	587 (9.8)	24 (11.4)	563 (9.7)	0.423
Peptic ulcer disease	1,577 (26.2)	58 (27.5)	1,519 (26.2)	0.674
Post-esophageal resection	27 (0.5)	2 (1.0)	25 (0.4)	0.270
Post-gastric resection	270 (4.5)	5 (2.4)	265 (4.6)	0.130
Drugs				
Anticancer drugs	43 (0.7)	2 (1.0)	41 (0.7)	0.683
Corticosteroids	404 (6.7)	56 (26.5)	348 (6.0)	<0.001
Prednisone	359 (6.0)	50 (23.7)	309 (5.3)	<0.001
Methylprednisolone	16 (0.3)	2 (1.0)	14 (0.2)	0.050
Betamethasone	9 (0.2)	1 (0.5)	8 (0.1)	0.215
Dexamethasone	10 (0.2)	1 (0.5)	9 (0.2)	0.264
Hydrocortisone	10 (0.2)	2 (1.0)	8 (0.1)	0.005
Prednisone-equivalent daily dose (mg)	10.7±18.6	15.5±20.5	9.94±18.2	0.047
NSAIDs	540 (9.0)	27 (12.8)	513 (8.8)	0.049
Acetaminophen	156 (2.6)	13 (6.2)	143 (2.5)	0.001
Proton pump inhibitor	1,678 (27.9)	76 (36.0)	1,602 (27.6)	0.008
Lansoprazole	793 (13.2)	34 (16.1)	759 (13.1)	0.202
Rabeprazole	301 (5.0)	12 (5.7)	289 (5.0)	0.645
Omeprazole	457 (7.6)	19 (9.0)	438 (7.6)	0.434
Esomeprazole	160 (2.7)	10 (4.7)	150 (2.6)	0.056
H2RA	468 (7.8)	21 (10.0)	447 (7.7)	0.232

CE, candida esophagitis; HIV, human immunodeficiency virus; IQR, interquartile range; HAART, highly active anti-retroviral therapy; HBV, hepatitis B virus; HCV, hepatitis C virus; NSAIDs, non-steroidal anti-inflammatory drugs; H2RA, H2-receptor antagonist.

Data are presented as number (%) or mean ±SD.

*p values for the comparison between patients with CE and those without CE.

**Table 2:** Upper gastrointestinal diseases concomitant with candida esophagitis evaluated by endoscopy with pathology.

Upper GI disease	All (n = 6,011)	CE (n = 211)	Non-CE (n = 5,800)	P*
Reflux esophagitis	651 (10.8)	15 (7.1)	636 (11.0)	0.077
Esophageal ulcer	33 (0.6)	5 (2.4)	28 (0.5)	<0.001
Esophageal advanced cancer	33 (0.6)	1 (0.5)	32 (0.6)	0.881
Esophageal malignancy	21 (0.4)	0 (0.0)	21 (0.4)	0.381
Gastric ulcer	271 (4.5)	16 (7.6)	255 (4.4)	0.028
Atrophic gastritis	3,030 (50.4)	115 (54.5)	2,915 (50.3)	0.226
Mild to moderate type	2199 (36.6)	70 (33.2)	2129 (36.7)	0.295
Severe type	669 (11.4)	38 (18.6)	631 (11.2)	0.001
Gastric advanced cancer	32 (0.5)	2 (1.0)	30 (0.5)	0.398
Gastric malignancy	174 (2.9)	9 (4.3)	165 (2.8)	0.227
Duodenal ulcer	100 (1.7)	5 (2.4)	95 (1.6)	0.414
Duodenal malignancy	78 (1.3)	4 (1.9)	74 (1.3)	0.434

GI, gastrointestinal; CE, candida esophagitis.

Data are presented as number (%).

*p values for the comparison between patients with CE and those with non-CE.

#### Univariate and multivariate odds ratios for CE

Risk factors for CE identified by multivariate analysis are shown in Table 3. Increasing age, HIV infection, and corticosteroid use were independently associated with CE and smoker and acetaminophen were marginally associated with CE, but sex, alcohol consumption, HCV infection, syphilis, post-gastric resection, and use of NSAIDs or PPIs were not.

**Table 3:** Risk factors for candida esophagitis (n = 6,011).

Factors	Crude OR	P	Adjusted OR	P
Age, years	1.00 (0.99–1.01)	0.504	1.02 (1.00–1.03)	0.007
Sex, male	1.19 (0.90–1.58)	0.216	1.08 (0.76–1.54)	0.651
Alcohol	0.75 (0.57–0.98)	0.038	0.87 (0.64–1.17)	0.351
Smoker	1.30 (0.98–1.71)	0.064	1.32 (0.96–1.81)	0.085
HIV infection	4.18 (2.98–5.86)	<0.001	4.92 (3.11–7.79)	<0.001
HBV infection	0.64 (0.20–2.04)	0.451	NA	NA
HCV infection	1.80 (1.12–2.89)	0.015	1.40 (0.85–2.31)	0.184
Syphilis	3.42 (2.06–5.69)	<0.001	1.32 (0.73–2.39)	0.356
Diabetes mellitus	0.89 (0.58–1.36)	0.595	NA	NA
Cardiovascular disease	1.03 (0.61–1.73)	0.911	NA	NA
Cerebrovascular disease	1.02 (0.14–7.53)	0.986	NA	NA
Chronic kidney disease	0.72 (0.30–1.74)	0.760	NA	NA
Liver cirrhosis	1.20 (0.77–1.85)	0.419	NA	NA
Peptic ulcer disease	1.20 (0.79–1.82)	0.674	NA	NA
Post-esophageal resection	2.21 (0.52–9.39)	0.270	NA	NA
Post-gastric resection	0.51 (0.21–1.24)	0.137	0.61 (0.25–1.52)	0.291
Anticancer drugs	1.34 (0.32–5.59)	0.684	NA	NA
Corticosteroids	5.66 (4.09–7.83)	<0.001	5.90 (4.10–8.50)	<0.001
NSAIDs	1.51 (1.00–2.29)	0.050	1.08 (0.69–1.67)	0.745
Acetaminophen	2.60 (1.45–4.66)	0.001	1.70 (0.91–3.16)	0.097
Proton pump inhibitor	1.48 (1.11–1.97)	0.008	1.11 (0.80–1.53)	0.532
H2RA	1.32 (0.83–2.10)	0.233	NA	NA

OR, odds ratio; HIV, human immunodeficiency virus; HBV, hepatitis B virus; HCV, hepatitis C virus; NSAIDs, non-steroidal anti-inflammatory drugs; H2RA, H2-receptor antagonists; NA, not applicable.

Values in parentheses represent 95% confidential intervals.

The risk factors identified by multivariate analysis for HIV-infected and non-HIV-infected patients are shown in Table 4. In HIV-infected patients, CD4 cell count<100μL significantly increased the risk of CE and history of HAART significantly decreased the risk. Among HIV-infected patients, 25% of the CE patients had CD4 cell counts >400/μL. In non-HIV-infected patients, increasing age and corticosteroid use were independently associated with CE, and HCV infection, peptic ulcer disease, and post-esophageal resection were marginally associated. Among corticosteroid users, higher prednisone-equivalent daily corticosteroid doses significantly increased the risk for CE (Table 5).

**Table 4:** Risk factors for candida esophagitis between patients with HIV infection (n = 430) and those without HIV infection (n = 5,581).

**HIV-infected patients (n = 430)**					
**Factors**	**Case/ Control (48/382)**	**Crude OR**	**P**	**Adjusted OR**	**P**
Age, years	46.0 ± 12.1 / 46.8 ± 11.6	0.99 (0.97–1.02)	0.678	1.02 (0.99–1.05)	0.138
Sex, male	47 / 362	2.60 (0.34–19.80)	0.357	2.89 (0.29–29.1)	0.369
Alcohol	24 / 212	0.80 (0.44–1.46)	0.471	NA	NA
Smoker	31 / 231	1.19 (0.64–2.23)	0.582	NA	NA
HBV infection	2 / 29	0.52 (0.12–2.29)	0.387	NA	NA
HCV infection	7 / 60	0.92 (0.39–2.14)	0.840	NA	NA
Syphilis	14 / 100	1.16 (0.60–2.23)	0.659	NA	NA
Diabetes mellitus	3 / 33	0.72 (0.21–2.45)	0.600	NA	NA
Cardiovascular disease	2 /11	1.49 (0.32–6.94)	0.609	NA	NA
Cerebrovascular disease	0 / 1	NA	NA	NA	NA
Chronic kidney disease	0 / 13	NA	NA	NA	NA
Liver cirrhosis	13 / 114	0.89 (0.45–1.75)	0.732	NA	NA
Peptic ulcer disease	3 / 64	0.33 (0.10–1.10)	0.059	0.36 (0.10–1.31)	0.120
Post-esophageal resection	0 / 0	NA	NA	NA	NA
Post-gastric resection	1 / 3	2.69 (0.27–26.4)	0.396	NA	NA
Anticancer drugs	1 / 5	1.60 (0.18–14.03)	0.669	NA	NA
Corticosteroids	10 / 17	5.65 (2.42–13.21)	<0.001	2.47 (0.79–7.76)	0.121
NSAIDs	7 / 46	1.25 (0.53–2.94)	0.614	NA	NA
Acetaminophen	4 / 22	1.49 (0.49–4.52)	0.483	NA	NA
Proton pump inhibitor	5 / 49	0.79 (0.30–2.09)	0.635	NA	NA
H2RA	6 / 16	3.27 (1.21–8.80)	0.019	2.61 (0.83–8.25)	0.102
MSM	37 / 259	1.60 (0.79–3.24)	0.194	1.25 (0.57–2.75)	0.581
CD4 cell count <100/μL	23 / 42	7.45 (3.89–14.28)	<0.001	4.83 (2.23–10.43)	<0.001
History of HAART	24 / 318	0.19 (0.10–0.35)	<0.001	0.35 (0.17–0.74)	0.006
**Non-HIV-infected patients (n = 5,581)**					
**Factors**	**Case/ Control (163/5,418)**	**Crude OR**	**P**	**Adjusted OR**	**P**
Age, years	64.5 ± 13.1 / 60.5 ± 13.9	1.02 (1.01–1.04)	<0.001	1.02 (1.01–1.03)	0.008
Sex, male	81 / 2,907	0.85 (0.62–1.17)	0.318	1.01 (0.79–1.56)	0.557
Alcohol	73 / 2,876	0.72 (0.52–0.98)	0.037	0.97 (0.69–1.37)	0.852
Smoker	79 / 2,417	1.17 (0.85–1.59)	0.330	NA	NA
HBV infection	1 / 98	0.34 (0.05–2.42)	0.255	NA	NA
HCV infection	13 / 259	1.72 (0.97–3.08)	0.065	1.70 (0.94–3.09)	0.081
Syphilis	4 / 54	2.50 (0.89–6.98)	0.081	2.33 (0.81–6.73)	0.116
Diabetes mellitus	22 / 725	1.00 (0.63–1.58)	0.997	NA	NA
Cardiovascular disease	14 / 417	1.12 (0.64–1.96)	0.684	NA	NA
Cerebrovascular disease	1 / 26	1.28 (0.17–9.49)	0.809	NA	NA
Chronic kidney disease	5 / 173	0.92 (0.41–2.07)	0.979	NA	NA
Liver cirrhosis	11 / 449	0.80 (0.43–1.48)	0.477	NA	NA
Peptic ulcer disease	55 / 1,455	1.39 (1.00–1.93)	0.051	1.38 (0.98–1.94)	0.061
Post-esophageal resection	2 / 25	2.68 (0.63–11.41)	0.165	3.72 (0.85–16.31)	0.082
Post-gastric resection	4 / 262	0.50 (0.18–1.35)	0.168	0.52 (0.19–1.42)	0.199
Anticancer drugs	1 / 36	0.92 (0.13–6.77)	0.937	NA	NA
Corticosteroids	49 / 363	5.99 (4.21–8.51)	<0.001	5.80 (3.90–8.63)	<0.001
NSAIDs	20 / 467	1.48 (0.92–2.39)	0.106	1.06 (0.64–1.77)	0.816
Acetaminophen	9 / 121	2.56 (1.28–5.13)	0.008	1.85 (0.88–3.87)	0.104
Proton pump inhibitor	71 / 1,553	1.92 (1.40–2.63)	<0.001	1.16 (0.82–1.65)	0.388
H2RA	15 / 431	1.17 (0.68–2.01)	0.563	NA	NA

OR, odds ratio; HIV, human immunodeficiency virus; HBV, hepatitis B virus; HCV, hepatitis C virus; NSAIDs, non-steroidal anti-inflammatory drugs; H2RA, H2-receptor antagonists; MSM, men who have sex with men; HAART, highly active anti-retroviral therapy; NA, not applicable.

Values are number or mean ±SD. Values in parentheses are 95% confidential intervals.

**Table 5:** Effect of prednisone-equivalent daily corticosteroid dose on risk for candida esophagitis in steroid users (n = 402*).

Prednisone-equivalent daily dose (mg)	Case/ control	Crude OR	P	P for trend	Age- and sex- adjusted OR	P	P for trend
<5 (n = 88)	8/80	Referent			Referent		
5–9 (n = 160)	17/143	1.19 (0.49–2.88)	0.701		1.18 (0.49–2.86)	0.714	
10–19 (n = 92)	16/76	2.11 (0.85–5.20)	0.107		2.06 (0.82–5.14)	0.122	
≥20 (n = 62)	15/47	3.19 (1.26–8.09)	0.015	0.028	3.09 (1.20–7.97)	0.019	0.043

OR, odds ratio.

*Of 404 corticosteroid users, 2 were eliminated because of insufficient data.

Values in parentheses are 95% confidential intervals.

## Discussion

In this study, we identified that, over the 13-year period from 2002 to 2014, CE prevalence showed significant increases in non-HIV-infected patients but decreases in HIV-infected patients. Moreover, risk analysis revealed that increasing age, HIV infection, and corticosteroids use were independently associated with CE, but alcohol, other infections, diabetes, anticancer drugs, and antisecretory drug use were not. Among HIV-infected patients, low CD4 cell count and absence of HAART were independent risk factors for CE. In corticosteroid users, higher prednisone-equivalent daily corticosteroid dose significantly increased CE risk.

CE prevalence in non-HIV-infected patients is reported to 0.3–7.3% [5,8,10,22,23], which is consistent with our findings of 1.5% in non-HIV-infected patients. Similarly for HIV-infected patients, CE prevalence was reported to be 42.8–51.8% in the pre-HAART era [24,25] and 8.5–16.7% in the HAART era [17,24], with which our finding of 9.8% prevalence in HIV-infected patients, most of whom received HAART (80.3%), concurs.

Little information about CE trends has been available to date, and most studies have focused on HIV-infected patients [12,24]. Declining CE incidence has been reported in this patient population by Mocroft et al [12], from 5.7 person-years in 1994 to 0.5 person-years in 2004, and Nkuize et al [24], from 42.8% in 1991–1994, 23.5% in 1999–2002, and 16.7% in 2005–2008. Likewise, CE prevalence decreased from 13.6% in 2002–2003 to 9.0% in 2012–2014 in the present study. This is probably due to the introduction of HAART, which helped avoid immunodeficiency, and the development of effective prophylaxis against opportunistic infection [26]. In contrast, CE prevalence in non-HIV-infected patients showed a significant increase over the same period, likely because, in a rapidly aging society, more people have comorbidities and take immunosuppressants [27,28], which can lead to the development of CE.

Several risk factors for CE have been investigated in case-control studies [6–8,10], although these studies had a small number of CE cases (three studies; n = 56 [6–8], one study; n = 163 [10]) and retrospective in nature. They identified corticosteroid or PPIs use, heavy drinking, uncontrolled DM, and cancer as CE risk factors. In our larger scale study investigating the very same factors, multivariate analysis similarly identified systemic corticosteroid use but also revealed increasing age and HIV infection as risk factors for CE.

In terms of systemic corticosteroid use, an association was found in non-HIV-infected patients in this study. This could be explained by the suppression of macrophages and polymorphonuclear leucocytes by glucocorticoids, which can in turn lead to the development of CE [29]. Moreover, Heidenreich et al [30] showed that glucocorticoids principally affect the capacity of monocytes to control extracellular growth of Candida species by inhibiting tumor necrosis factor alpha secretion. We also found that higher prednisone-equivalent dose of corticosteroids was a risk factor for CE. Among the very few studies to date that have investigated the association between opportunistic infection and steroid dose, Yale et al [31] demonstrated that >30 mg of steroid daily was further associated with worsened *Pneumocystis carinii* pneumonia (PCP), compared with <30 mg daily, and Porges et al [32] demonstrated that >40 mg daily was associated with PCP development among patients with systemic lupus erythematosus. Our study is the first to investigate the association between steroid dose and CE and our findings suggest that patients who receive high-dose steroids have lower cellular immunity, which can lead to CE development.

Increasing age was identified as a risk factor for CE in non-HIV-infected patients in this study. It is possible that, in non-HIV-infected patients, increasing age leads to impaired immunity due to defects in the hematopoietic bone marrow and in peripheral lymphocyte migration, maturation, and function [9]. Another reason could be that increasing age causes a decline in cellular immunity of the epithelial layer, which can lead to colonization of *Candida* species [33].

HIV infection itself was a risk factor for CE in this study. We suggest that impaired oral immunity plays a role in this. Oral immunity may be affected earlier than immunity in other organs [34] and thus facilitate the development of CE early in HIV infection. Indeed, Chih-Ko et al [34] demonstrated that salivary function is impaired early in the course of HIV infection. In the present study, the analysis of HIV-infected subjects showed an association between CD4 cell count <100/μL and CE, although 25% of HIV-infected patients with CE also had a relatively high CD4 count (CD4>400). Buchacz et al found that the median CD4 cell count in CE-infected patients increased from 43/μL in the pre-HAART era (1994–1997) to 100/μL in the HAART era (2003–2007). These findings suggest that in the HAART era, HIV-infected patients are likely to develop CE even though their CD4 cell count is relatively high.

Previous studies have suggested that PPIs use is associated with CE because PPIs reduce gastric acidity, an important barrier for most microorganisms, and this can result in a number of infections [35]; however, the findings of this association are inconsistent. Chocarro et al [6] showed that, among 51 CE patients and 102 controls, PPIs use was independently associated with CE. In contrast, in case-control studies by Weerasuriya et al [7] and Choi et al [10], PPIs use was not associated with CE, consistent with our data. In clinical practice, PPIs are frequently administered when NSAIDs are used for protection from NSAID-induced peptic ulcer disease, but NSAID use was not adjusted for in previous studies that were also small in scale [6,7,10]. Therefore, a large-scale study with adjustment for NSAIDs is required to identify whether PPIs are a risk of CE.

The strengths of this study include its prospective nature, large scale, and evaluation of detailed HIV information, which was obtained by performing HIV testing in all subjects. Its limitations include the following facts. 1) Because sufficient information was not collected on the pre-endoscopy setting, we could not evaluate the potential risk factors of chronic obstructive pulmonary disease and antibiotic or herbal medication use. 2) Selection bias was present because endoscopy could not be performed for critically ill or intubated patients. 3) Endoscopy is not always performed for HIV-infected patients with oral candidiasis or esophageal symptoms who are empirically treated with antifungal therapy. Thus, this study potentially underestimated the number of CE cases. 4) This study has a cross-sectional design, so we can only comment on risk factors as being associated with disease but we cannot truly understand the causal pathways. 5) This case-control study is potentially affected by recall or interviewer bias, particularly for survey items on alcohol consumption, medication use, and smoking. Therefore, we attempted to limit bias by blinding survey results and performing surveys prior to endoscopy. 6) The community setting of the controls (undergoing endoscopy without CE) may have been different from that of cases (patients with CE); thus, unmeasured confounders exist. In particular, co-morbidities have a confounding effect. In addition, the study would have been better if subjects had been matched for age, sex, and co-morbidities rather than just adjusting the model for these factors.

In conclusion, this study identified that CE prevalence over a 13-year period increased in non-HIV-infected patients but decreased in HIV-infected patients. Besides increasing age, HIV infection itself and corticosteroid use increased the risk for CE development, but antisecretory drug use did not. In HIV-infected patients, a low CD4 count and absence of HAART were independent risk factors for CE. In corticosteroid users, CE was associated with higher prednisone-equivalent dose. Given that the elderly tend to have co-morbidities and take drugs, CE prevalence is expected to continue increasing in today’s rapid aging society.
